# IKKε and TBK1 expression in gastric cancer

**DOI:** 10.18632/oncotarget.9069

**Published:** 2016-04-28

**Authors:** Seung Eun Lee, Mineui Hong, Junhun Cho, Jeeyun Lee, Kyoung-Mee Kim

**Affiliations:** ^1^ Department of Pathology, Konkuk University School of Medicine, Konkuk University Medical Center, Seoul, Korea; ^2^ Department of Pathology, Hallym University Kangnam Sacred Heart Hospital, Seoul, Korea; ^3^ Department of Pathology and Translational Genomics, Samsung Medical Center, Sungkyunkwan University School of Medicine, Seoul, Korea; ^4^ Division of Hematology-Oncology, Department of Medicine, Samsung Medical Center, Sungkyunkwan University School of Medicine, Seoul, Korea

**Keywords:** IKKε, TBK1, gastric, cancer, therapy

## Abstract

Inhibitor of kappa B kinase epsilon (IKKε) and TANK-binding kinase 1 (TBK1) are non-canonical IKKs. IKKε and TBK1 share the kinase domain and are similar in their ability to activate the nuclear factor-kappa B signaling pathway. IKKε and TBK1 are overexpressed through multiple mechanisms in various human cancers. However, the expression of IKKε and TBK1 in gastric cancer and their role in prognosis have not been studied.

To investigate overexpression of the IKKε and TBK1 proteins in gastric cancer and their relationship with clinicopathologic factors, we performed immunohistochemical staining using a tissue microarray. Tissue microarray samples were obtained from 1,107 gastric cancer patients who underwent R0 gastrectomy with extensive lymph node dissection and adjuvant chemotherapy.

We identified expression of IKKε in 150 (13.6%) and TBK1 in 38 (3.4%) gastric cancers. Furthermore, co-expression of IKKε and TBK1 was identified in 1.5% of cases. Co-expression of IKKε and TBK1 was associated with differentiated intestinal histology and earlier T stage. In a multivariate binary logistic regression model, intestinal histologic type by Lauren classification and early AJCC stage were significant predictors for expression of IKKε and TBK1 proteins in gastric cancer. Changes in IKKε and TBK1 expression may be involved in the development of intestinal-type gastric cancer. The overexpression of IKKε and TBK1 should be considered in selected patients with intestinal-type gastric cancer.

In conclusion, this is the first large-scale study investigating the relationships between expression of IKKε and TBK1 and clinicopathologic features of gastric cancer. The role of IKKε and TBK1 in intestinal-type gastric cancer pathogenesis should be elucidated by further investigation.

## INTRODUCTION

Gastric cancer is the third leading cause of cancer-related death worldwide. Identifying effective pathway inhibition is a key aspect of the development of targeted therapeutics. The success of trastuzumab in HER2-positive gastric cancer patients has elicited efforts to discover new molecular targets in gastric cancer.

The nuclear factor-kappa B (NF-κB) pathway is a key regulator that activates transcription of genes involved the inflammatory immune response, proliferation, cell survival and cell invasion. Activation of NF-kB is frequently observed in solid tumors and hematological malignancies [1]. NF-κB complexes are retained in the cytoplasm by a family of NF-κB-binding proteins known as inhibitors of NF-κB (IκBs) [1]. Various stimulants trigger activation of the IKK (IκBs kinase complex), leading to proteasomal degradation of IκBs. Consequently, NF-κB dimers are released in the nucleus and induce transcription of target genes related to inflammation and survival.

The IKK family can be divided into two groups: canonical IKKs (IKKα, IKKβ and a nonenzymatic regulatory component, IKKγ/NEMO) and non-canonical IKKs (IKKε and TBK1) [2]. Although IKKε and TANK-binding kinase 1 (TBK1) are characterized as activators of NF-κB, they are not essential for NF-κB activation [3, 4]. Instead, IKKε and TBK1 play critical roles in antiviral response via phosphorylation and activation of transcription factors IRF3, IRF7 and STAT1 [1, 5]. Furthermore, these non-canonical IKKs are also involved in the survival, tumorigenesis and development of various cancers [6–8]. Although IKKε expression is restricted to particular tissues, such as lymphoid tissues, peripheral blood lymphocytes and the pancreas [9], TBK1 is constitutively expressed in many normal tissues, including the immune cells, brain, lungs, gastrointestinal tract, and reproductive organs [10]. IKKε is overexpressed through multiple mechanisms in various human cancers, such as breast, ovarian, and prostate cancer. IKKε overexpression in breast and ovarian cancer was increased due to amplification or unknown mutations regulating IKKε transcript levels [2, 11]. IKKε has been identified as an oncogene in breast [2] and ovarian cancer [11, 12] and is associated with poor prognosis [7, 13]. Recent reports suggested that overexpression of IKKε may play a role in tumorigenesis of prostatic and esophageal squamous cell carcinoma [14, 15]. TBK1’s role in cancer may be due to its involvement in regulation of cell growth and proliferation, angiogenesis and oncogenic transformation [8, 16–18]. Through functional genomics, Barbie et al. [6] identified that TBK1 was essential for KRAS mutant cancer cell lines. However, subsequent studies found no relationship between oncogenic KRAS and TBK1 [19].

Thus far, the expression of IKKε and TBK1 in gastric cancer and their role in prognosis have not been studied. To investigate overexpression of IKKε and TBK1 in gastric cancer and their relation to clinicopathologic factors, we performed immunohistochemical staining in 1,107 resected gastric cancers using a tissue microarray approach.

## RESULTS

### Expression of IKKε and TBK1 in gastric cancer

Expression of IKKε and TBK1 was observed in 13.6% (150/1107) and 3.4% (38/1107) of gastric cancer patients, respectively. Associations between IKKε and TBK1 expression and clinicopathological factors were evaluated (Table 1). IKKε and TBK1 expression were correlated with histologic differentiation and histologic type by Lauren classification. Differentiated tumors and intestinal-type gastric cancer by Lauren classification showed increased IKKε and TBK1 expression compared to undifferentiated and diffuse types. Expression of IKKε and TBK1 was associated with earlier AJCC stage (based on AJCC seventh edition; *p*=0.019 and *p=*0.003, respectively). Expression of IKKε was observed in 16.7%, 15.9% and 10.2% of stage I, II, and III tumors, respectively. Expression of TBK1 was observed in 8.3%, 4.1% and 1.7% of stage I, II, and III tumors, respectively.

**Table 1:** The association of IKK and TBK1 expression and clinicopathological factors

		IKK expression				TBK1 expression		
Total No. of cases	Positive	Negative	Positive	Negative				
n=1107 (%)	n=150 (13.6%)	n=957 (86.4%)	*P* -value	n=38 (3.4%)	n=1069 (96.6%)	*P* -value		
Gender					0.002			0.076
	Male	725 (65.5%)	115 (15.9%)	610 (84.1%)		30 (4.1%)	695 (95.9%)	
	Female	382 (34.5%)	35 (9.2%)	347 (90.8%)		8 (2.1%)	374 (97.9%)	
Age (years)					<0.001			0.058
	<60	792 (71.5%)	88 (11.1%)	704 (88.9%)		22 (2.8%)	770 (97.2%)	
	≥60	315 (28.5%)	62 (19.7%)	253 (80.3%)		16 (5.1%)	299 (94.9%)	
Tumor location					0.461			0.382
	Upper third	117 (10.6%)	18 (15.4%)	99 (84.6%)		1 (0.9%)	116 (99.1%)	
	Middle third	315 (28.5%)	36 (11.4%)	279 (88.6%)		10 (3.2%)	305 (96.8%)	
	Lower third	626 (56.5%)	87 (13.9%)	539 (86.1%)		25 (4.0%)	601 (96.0%)	
	Whole	49 (4.4%)	9 (18.4%)	40 (81.6%)		2 (4.1%)	47 (95.9%)	
Lauren classification					<0.001			<0.001
	Intestinal	319 (28.8%)	72 (22.6%)	247 (77.4%)		19 (6.0%)	300 (94.0%)	
	Diffuse	766 (69.2%)	70 (9.1%)	696 (90.9%)		16 (2.1%)	750 (97.9%)	
	Mixed	22 (2.0%)	8 (36.4%)	14 (63.6%)		3 (13.6%)	19 (86.4%)	
Histology					<0.001			0.001
	Differentiated	303 (37.4%)	70 (23.1%)	233 (76.9%)		19 (6.3%)	284 (93.7%)	
	Undifferentiated	804 (72.6%)	80 (10.0%)	724 (90.0%)		19 (2.4%)	785 (97.6%)	
T stage					0.081			0.011
	T1	108 (9.8%)	19 (17.6%)	89 (82.4%)		9 (8.3%)	99 (91.7%)	
	T2	124 (11.2%)	22 (17.7%)	102 (82.3%)		6 (4.8%)	118 (95.2%)	
	T3	686 (62.0%)	92 (13.4%)	594 (86.6%)		20 (2.9%)	666 (97.1%)	
	T4	189 (17.1%)	17 (9.0%)	172 (91.0%)		3 (1.6%)	186 (98.4%)	
N stage					0.109			0.233
	N0	102 (9.2%)	10 (9.8%)	92 (90.2%)		3 (2.9%)	99 (97.1%)	
	N1	558 (50.4%)	89 (15.9%)	469 (84.1%)		25 (4.5%)	533 (95.5%)	
	N2	289 (26.1%)	35 (12.1%)	254 (87.9%)		8 (2.8%)	281 (97.2%)	
	N3	158 (14.3%)	16 (10.1%)	142 (89.9%)		2 (1.3%)	156 (98.7%)	
AJCC 7^th^ stage					0.019			0.003
	I	96 (8.7%)	16 (16.7%)	80 (83.3%)		8 (8.3%)	88 (91.7%)	
	II	540 (48.8%)	86 (15.9%)	454 (84.1%)		22 (4.1%)	518 (95.9%)	
	III	471 (42.5%)	48 (10.2%)	423 (89.8%)		8 (1.7%)	463 (98.3%)	
Recurrence of disease					0.029			0.356
	Yes	429 (38.8%)	46 (10.7%)	383 (89.3%)		12 (2.8%)	417 (97.2%)	
	No	678 (61.2%)	104 (15.3%)	574 (84.7%)		26 (3.8%)	652 (96.2%)	
Death of disease					0.128			0.544
	Yes	401 (36.2%)	46 (11.5%)	355 (88.5%)		12 (3.0%)	389 (97.0%)	
	No	706 (63.8%)	104 (14.7%)	602 (85.3%)		26 (3.7%)	680 (96.3%)	

There was a significant association between IKKε and TBK1 expression (*p*<0.001), as 97.8% (936/957) of tumors with IKKε negativity showed negative expression of TBK1 (Table 2).

**Table 2:** Association between the IKK and TBK1 expression

Variable	IKK expression		
	Positive (n=150)	Negative (n=957)	*P* - value
TBK1 expression			<0.001
Positive (n=38)	17 (11.3%)	21 (2.2%)	
Negative (n=1069)	133 (88.7%)	936 (97.8%)	

### Co-expression of IKK and TBK1 in gastric cancer

**Figure 1:**
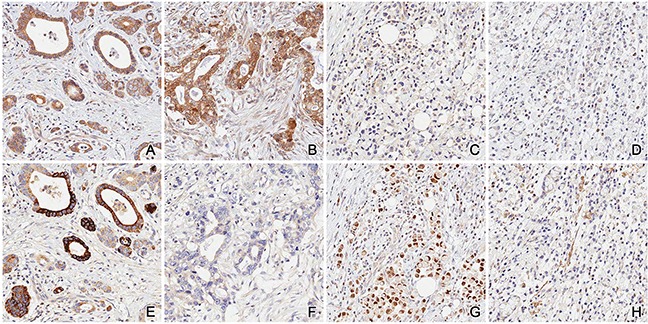
Representative examples of the four subgroups **A**. & **E**. IKK+/TBK1+; **B**. & **F**. IKK+/TBK1-; **C**. & **G**. IKK-/TBK1+; **D**. & **H**. IKK-/TBK1-.

We classified IKKε and TBK1 expression status into four subgroups as follows: IKKε-/TBK1- (*n*=936, 84.6%); IKKε+/TBK1- (*n*=133, 12.0%); IKKε-/TBK1+ (*n*=21, 1.9%); IKKε+/TBK1+ (*n*=17, 1.5%) (Figure 1). Clinicopathological characteristics among these four groups were also evaluated (Table 3). Intestinal-type gastric cancer on Lauren classification and differentiated tumors were more frequent in the IKKε+/TBK1+ subgroup than in the IKKε-/TBK1-subgroup (*p*<0.001 and *p*<0.001, respectively). Although N stage was not significantly associated with expression of IKKε and TBK1, patients in the IKKε+/TBK1+ subgroup were more likely to have earlier T stage and lower AJCC stage than those in the IKKε-/TBK1-subgroup (*p*=0.011 and *p=*0.002, respectively).

**Table 3:** The association of co-expressions of IKK and TBK1 and clinicopathological factors

		IKK-/TBK1-	IKK+/TBK1-	IKK-/TBK1+	IKK+/TBK1+	*P* – value
N=936 (84.6%)	N=133 (12.0%)	N=21 (1.9%)	N=17 (1.5%)			
Gender						0.01
	Male	594 (81.9%)	101 (13.9%)	16 (2.2%)	14 (1.9%)	
	Female	342 (89.5%)	32 (8.4%)	5 (1.3%)	3 (0.8%)	
Age (years)						0.001
	<60	691 (87.2%)	79 (10.0%)	13 (1.6%)	9 (1.1%)	
	≥60	245 (77.8%)	54 (17.1%)	8 (2.5%)	8 (2.5%)	
Tumor location						0.382
	Upper third	99 (84.6%)	17 (14.5%)	0 (0%)	1 (0.9%)	
	Middle third	275 (87.3%)	30 (9.5%)	4 (1.3%)	6 (1.9%)	
	Lower third	524 (83.7%)	77 (12.3%)	15 (2.4%)	10 (1.6%)	
	Whole	38 (77.6%)	9 (18.4%)	2 (4.1%)	0 (0%)	
Lauren classification						<0.001
	Intestinal	238 (74.6%)	62 (19.4%)	9 (2.8%)	10 (3.1%)	
	Diffuse	685 (89.4%)	65 (8.5%)	11 (1.4%)	5 (0.7%)	
	Mixed	13 (59.1%)	6 (27.3%)	1 (4.5%)	2 (9.1%)	
Histology						<0.001
	Differentiated	225 (74.3%)	59 (19.5%)	8 (2.6%)	11 (3.6%)	
	Undifferentiated	711 (88.4%)	74 (9.2%)	13 (1.6%)	6 (0.7%)	
T stage						0.011
	T1	85 (78.7%)	14 (13.0%)	4 (3.7%)	5 (4.6%)	
	T2	100 (80.6%)	18 (14.5%)	2 (1.6%)	4 (3.2%)	
	T3	581 (84.7%)	85 (12.4%)	13 (1.9%)	7 (1.0%)	
	T4	170 (89.9%)	16 (8.5%)	2 (1.1%)	1 (0.5%)	
N stage						0.069*
	N0	90 (88.2%)	9 (8.8%)	2 (2.0%)	1 (1.0%)	
	N1	457 (81.9%)	76 (13.6%)	12 (2.2%)	13 (2.3%)	
	N2	248 (85.8%)	33 (11.4%)	6 (2.1%)	2 (0.7%)	
	N3	141 (89.2%)	15 (9.5%)	1 (0.6%)	1 (0.6%)	
AJCC 7^th^ stage						0.002
	I	76 (8.1%)	12 (9.0%)	4 (19.0%)	4 (23.5%)	
	II	444 (47.4%)	74 (55.6%)	10 (47.6%)	12 (70.6%)	
	III	416 (44.4%)	47 (35.3%)	7 (33.3%)	1 (5.9%)	
Recurrence of disease						0.101
	Yes	374 (87.2%)	43 (10.0%)	9 (1.8%)	3 (0.7%)	
	No	562 (82.9%)	90 (13.3%)	12 (2.1%)	14 (2.1%)	
Death of disease						0.258
	Yes	346 (86.3%)	43 (10.7%)	9 (2.2%)	3 (0.7%)	
	No	590 (83.6%)	90 (12.7%)	12 (1.7%)	14 (2.0%)	

In univariate binary logistic regression analysis, several clinicopathologic variables were related to expression of IKKε+/TBK1+, including intestinal and differentiated histologic types, earlier T stage, and earlier AJCC stage. Among these variables, histology type and AJCC stage were significant predictors in multivariate analysis. The adjusted odds ratio (OR) of differentiated histology was 4.579, with a 95% CI 1.669-12.566 when compared with undifferentiated histology. The ORs of AJCC stage I and stage II were 18.914 and 9.935 (95% CI, 1.995-165 and 1.283-76.913) when compared with stage III (Table 4).

**Table 4:** Multivariate analysis of clinicopathological factors for co-expression of IKK/TBK1 in gastric cancer

	Odds ratio	95% CI	*P* - value
Histology			0.003
Undifferentiated	1.000		
Differentiated	4.579	1.669 - 12.566	
AJCC 7th stage			0.037
Stage I	18.194	1.995 - 165.910	0.010
Stage II	9.935	1.283 - 76.913	0.028
Stage III	1.000		

### Prognostic significance of IKK and TBK1 co-expression in gastric cancer

Overall, the mean follow-up period was 79.8, 73.8 and 54.6 months in AJCC stage I, II, and III, respectively. During the follow-up periods, 10.4%, 25.4% and 59.9% of patients in stage I, II and III had a recurrence and 8.3%, 22.8% and 57.3% of patients in stage I, II and III died of their disease.

**Figure 2:**
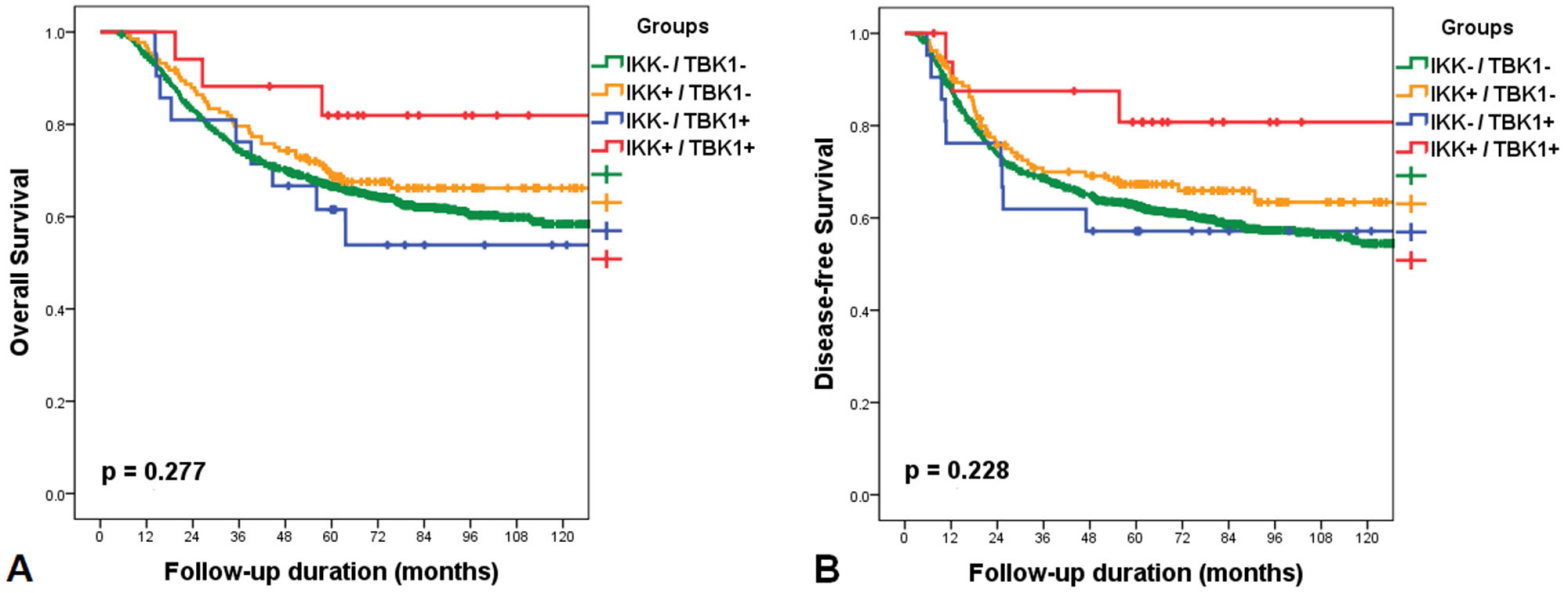
Kaplan-Meier survival curves with log-rank test of overall survival after classification into the following four subgroups: IKK-/TBK1-; IKK+/TBK1-; IKK-/TBK1+; IKK+/TBK1+ The survival curve of the IKK+/TBK1+ subgroup was different from those of other groups, but this difference was not statistically significant.

Patients in the IKKε+/TBK1+ subgroup showed longer overall survival (mean=114.7 months; 95% CI 107.3-118.9) than those in the IKKε-/TBK1- subgroup (mean=113.2 months; 95% CI 108.7-117.8; *p*=0.125), IKKε+/TBK1- subgroup (mean=118.1 months; 95% CI 107.4-128.7; *p*=0.235), and IKKε-/TBK1+ subgroup (mean=98.1 months; 95% CI 71.5-124.7; *p*=0.094). The IKKε+/TBK1+ subgroup showed longer disease-free survival (mean 125.8 months; 95% CI 101.5-150.0) than the IKKε-/TBK1- subgroup (mean=105.1 months; 95% CI 100.1-110.1; *p*=0.104), IKKε +/TBK1- subgroup (mean=104.6 months; 95% CI 93.7-115.5; *p*=0.240), and IKKε-/TBK1+ subgroup (mean=94.6 months; 95% CI 66.1-123.0; *p*=0.110). When survival curves were compared by log-rank test in Kaplan-Meier survival analyses, there were no survival differences in relation to expression of IKKε or TBK1. However, the survival curve of the IKKε+/TBK1+ subgroup varied from those of other groups, although this did not reach statistical significance due to the small number of events in the IKKε+/TBK1+ subgroup (Figure 2).

## DISCUSSION

Our study sought to investigate IKKε and TBK1 expression in gastric cancer and their role in prognosis. We identified IKKε and TBK1 expression in 13.6% and 3.4% of gastric cancers, respectively, and co-expression of IKKε and TBK1 in 1.5% of cases. Our findings suggest that IKKε and TBK1 may be good molecular target candidates, warranting future study to elucidate the underlying common regulatory mechanism.

Identifying specific biomarkers for patient selection and effective pathway inhibition is a key element in the development of targeted therapies. In particular, kinase oncogenes may be attractive therapeutic targets. IKKε and TBK1 are serine/threonine protein kinases belonging to the IKK family. Although IKKε and TBK1 exhibit differential expression patterns, they share the kinase domain and are similar in their ability to activate the NF-kB signaling pathway. NF-kB signaling pathway activation may be related to distinct mechanisms in different tumor types. Basically, NF-kBs affect cell survival and proliferation in cancer by inducing expression of genes coding for key anti-apoptotic proteins, such as Bcl-2 and IAP-1/2 and mitogenic genes, such as myc and cyclin-D. Hence, the function of the NF-kB signaling pathway is to protect cancer cells from apoptosis and drive their proliferation. Finally, IKKε and TBK1 are kinase oncogenes. Previous studies revealed that IKKε and TBK1 play a significant role in several cancers. Boehm et al. showed that IKKε is amplified and overexpressed in a breast cancer cell line and human breast cancer tissue [7]. Guo et al. recently showed that overexpression of IKKε in ovarian cancer was associated with late-stage and high-grade tumors [11]. Recently, Deng et al. [23] reported that patients with HER2-positive breast cancer may benefit from anti-TBK1/IKKε plus anti-HER2 combination therapies. TBK1/IKKε inhibition promoted cellular senescence by suppressing p65–NF-kB and inducing p16Ink4a. Although IKKε is not essential for growth of mouse Her2/Neu tumor cells, shRNA-mediated knockdown of TBK1 alone efficiently inhibited growth of both mouse and human HER2-positive breast cancer cells. Thus, TBK1 could be critical for survival and growth of tumors with HER2 amplification [23].

The oncogenic potential of IKKε and TBK1 indicate these proteins to be possible therapeutic targets. TBK1/IKKε inhibitors have shown low specificity, as they have multiple targets such as PDK1, JNK and p38 MAP kinases [24, 25]. Recently, Reilly et al. [26] discovered a small molecule inhibitor of IKKε and TBK1 kinases called amlexanox, which has been shown to selectively inhibit both IKKε and TBK1.

In our study, the multivariate binary logistic regression model was applied to determine prediction factors for IKKε+/TBK1+ expression in gastric cancer. Multivariate analysis showed that tissues with differentiated histology and earlier AJCC stage were correlated with IKKε+/TBK1+ expression. The frequency of IKKε and TBK1 co-expression was relatively high in early T stage, suggesting that alteration of IKKε and TBK1 could be more involved in the development of gastric cancer. Furthermore, co-expression of IKKε and TBK1 was associated with more differentiated histology, namely, intestinal-type gastric cancer. In this study, patients in the IKKε+/TBK1+ subgroup had a longer life span. Co-expression of IKKε and TBK1 was more frequent in early T stage tumors and those with more differentiated (intestinal-type) histology, which might have been related to good prognosis.

Recent studies have classified four major genomic groups of gastric cancer on a molecular and genomic basis: EBV-infected tumors, those with microsatellite instability, genomically-stable tumors, and those with chromosomal instability. Chromosomal-instability tumors were of the intestinal histology type [27]. Recently, we also classified gastric cancer into four molecular subtypes, which are closely associated with distinct clinical outcomes [28]. However, traditionally, gastric cancer is divided into two main subtypes on the basis of Lauren classification–intestinal and diffuse. These subtypes have different molecular pathogenesis. In the intestinal type, multistep progression initiated by *Helicobacter pylori* infection is associated with pathogenesis. Preferentially altered genes include*KRAS* and *HER2*, which are overexpressed in about 20% of gastric cancer [29–32]. Diffuse-type gastric cancer does not arise from step-wise progression and is associated with loss of cell cohesion due to biallelic inactivation of *CDH1*. Sporadically altered genes include *BCL2* and *FGFR2* in diffuse-type gastric carcinomas [33–36]. We demonstrated that alteration of IKKε and TBK1, albeit small, may be involved in the pathogenesis of intestinal-type gastric cancer. Thus, testing for IKKε and TBK1 overexpression should be considered in certain patients, such as those with intestinal-type gastric cancer.

We did not observe statistically significant survival differences between the four IKKε and TBK1 expression subgroups. However, the survival curve of the IKKε+/TBK1+ subgroup varied compared to other groups. This finding may be due to the small number of events in the IKKε+/TBK1+ subgroup. Our results do not agree with those of several previous studies. This discrepancy could be due to the differing roles of *IKKε* and *TBK1* in gastric cancer or the small number of positive cases. Further investigation is needed to determine its role in gastric carcinogenesis.

To the best of our knowledge, this is the first large-scale study investigating the relationship between the expression of IKKε and TBK1 and clinicopathologic features of gastric cancer. We determined the expression of IKKε and TBK1, and co-expression of IKKε and TBK1 was associated with differentiated intestinal histology and earlier tumor stage. The role of IKKε and TBK1 in intestinal-type gastric cancer pathogenesis should be elucidated by further study.

## MATERIALS AND METHODS

### Patients

Gastric cancer tissue samples were retrospectively collected from 1,107 patients (stages IB to IVa) who underwent R0 gastrectomy with extensive node dissection (D2) and adjuvant chemoradiation therapy (INT-0116 regimen) [20, 21] from 2000 to 2008 at Samsung Medical Center in Seoul, Korea. Clinicopathological characteristics obtained from medical records included sex, age, tumor size, tumor location, histological type, Lauren classification, and differentiation grade. Tumor histology was classified into 2 groups: differentiated, which included well- or moderately-differentiated tubular and papillary adenocarcinomas, and undifferentiated, which included poorly-differentiated adenocarcinomas and signet ring cell carcinomas.

### Immunohistochemistry

For tissue microarray, we reviewed all H&E-stained slides and representative histological areas were carefully selected and marked on paraffin blocks. From each paraffin block, four primary gastric cancer tissue cores (diameter = 0.6 mm) were taken from the invasive front, both lateral sides, and the luminal surface area of the tumor using AccuMax (IsuAbxis, Seoul, Korea) as previously described [22]. Immunohistochemistry was performed on formalin-fixed, paraffin-embedded, 4-μm-thick tissue sections using rabbit monoclonal antibody IKKε (D20G4, Cell Signaling Technology, Danvers, MA, USA, 1:50 dilution) and TBK1/NAK (D1B4, Cell Signaling Technology, Danvers, MA, USA, 1:200 dilution). For IKKε, we incubated primary antibody overnight at 4°C and used a DAKO Envision™ Detection Kit (DAKO, Glostrup, Denmark) for 30 minutes. For TBK1, we incubated primary antibody for 15 minutes with Bond-max autoimmunostainer (Leica Biosystem, Melbourne, Australia) using Bond™ Polymer refine detection (DS9800, Vision Biosystems, Melbourne, Australia) according to the manufacturer’s protocol. For the interpretation of IKKε and TBK1 Immunohistochemistry, strong, distinct cytoplasmic staining with membranous accentuation was considered positive.

### Statistical analysis

Statistical analysis was performed by SPSS 19.0 for Windows (SPSS, Chicago, IL, USA). Categorical variables were compared using Pearson’s chi-squared test or Fisher’s exact test, and continuous variables, which are presented as means ± SD, using the t-test. Factors found to be significant (*p*<0.05) in univariate analysis were included in subsequent multivariate logistic regression analysis to identify independent variables associated with IKK and TBK1 expression. Disease-free survival was defined as the time from surgery to first relapse. The Kaplan–Meier method was used to calculate disease-free and overall survival, and survival curves were compared by log-rank test. All tests were two sided, and *p* values <0.05 were considered statistically significant.
